# Leçons faisant Partie du Cours de Médecine Legale

**Published:** 1821-07

**Authors:** 


### CRITICAL ANALYSES

OF

### RECENT PUBLICATIONS, IN THE DIFFERENT BRANCHES OF MEDICINE AND SURGERY,

### In the Literature of Foreign Nations.

---

Πατρίδες ἄρα
Ανδράσιν, οὐ πατραῖς ἄνδρες, ἀγαλλόμεθα.

---

*Leçons faisant Partie du Cours de Médecine Legale de* M. Orfila, Professeur de Medecine légale à la Faculté de Médecine de Paris, &c. &c. 8vo. pp. 487; orné de vingt-deux planches, dont sept colorées. Bechet, jeune. Paris, 1821.

IT is the part of the author's Lectures on Medical Jurisprudence that relates to *Poisons* of which this work is constituted. Of Prof. Orfila's peculiar abilities for the illustration of this subject, it is unnecessary for us to speak: his *Toxicology* had already presented us with knowledge that contrasted, in a remarkable manner, with the vague information respecting the effects of the greater part of poisons on the animal economy that had hitherto been promulgated. But, though we had devoted much time to the application of the facts related in that work to the views of forensic medicine, we still find much original and interesting matter in the Lectures before us; and the author, by a new arrangement of his subjects, has here much facilitated the acquisition of the desired knowledge.

There are but very few instances where we can affirm, from observation of the symptoms during life, or the lesions of tissue found after death, what particular poison may have been administered: the great utility, in respect to the purposes of forensic inquiry, of knowledge of the general effects of poisons, is in its furnishing us with such suggestions as may lead us at once to researches qualified to furnish more valid evidence. Descriptions of a general kind are here, then, admissible, and qualified to prevent the necessity of as many particular descriptions as there are distinct species of poison. The author has, therefore, collected into groups such substances as produce nearly similar symptoms and lesions of tissue; giving a description appertaining to them generally, and then, on noticing each poison particularly, he has mentioned the effects which may be peculiar to the several individuals of the class. This method leads him to make some statements which are not, in all probability, strictly true, or sufficiently precise for the purposes of physiology; as, for example, when he says that the general symptoms produced by iodine are similar to those which arise from the action of the concentrated mineral acids, and that those symptoms do not depend on the absorption of the iodine, but merely on the local irritation excited by it. Now it seems reasonable to infer that there is something peculiar in the general symptoms of the effects of iodine, and that this substance is absorbed and produces effects dependent on such absorption; or how could it cure bronchocele, which the concentrated acids would not cure? But these peculiarities, if they exist, cannot be recognized in the phenomena which we have to regard in our researches for forensic evidence: the effects we witness and are able to appreciate, are such as depend on the irritative properties of the substance, and they are commensurate, in their severity, with the local disorganization produced; just as it happens with the concentrated acids and several other corrosive substances. It is desirable, certainly, that we could know from the symptoms what peculiar poison had produced them, but it does not seem probable that our knowledge will ever arrive at such a state of perfection: it is, at least, very far from this perfection at present, and hence we are enabled to form only conjectures from the character of the symptoms, that are, however, in many instances, sufficiently accurate to direct us at once to researches proper for the discovery of the cause. In order, then, that we may know that poisoning has occurred from any given substance, it is necessary for us to be acquainted, 1°, with the physical characters of the poisonous substance; 2°, (when it is of the mineral kind,) with the chemical experiments proper for demonstrating its presence, whether it be pure or mingled or combined with matters which conceal it; 3°, with the symptoms and the alterations of tissue which it produces; and, 4°, with its mode of action on the animal economy. It is in each of these points of view that the author has considered the several poisons noticed in this work; and, in addition to his verbal descriptions, he has attached to it several plates representing the chief of the most common poisonous plants which are indigenous to Europe, the viper, and the venomous insects which are productive of serious effects on man. Six of these plates (besides that of the insects,) are coloured, and represent the poisonous champignons and fungi: these, especially, constitute a particularly valuable addition to the work. The descriptions of the other vegetables, of which plates are given, are by Dr. A. Richard, and they excel in perspicuity and minute precision any hitherto extant: they comprise, indeed, several original observations, on the structure of the seed-vessels chiefly, (of which diagrams are given,) that will be interesting to botanists.

By passing over what may be considered adventitious and elementary instruction,—consisting in the botanical and chemical descriptions, and such experiments for the detection of mineral substances as are not peculiar to the author, and do not vary from the *common* processes of chemical analysis,—we shall so far reduce the work as to enable us to give a tolerably comprehensive account of the rest in an extent of space not incongruous with the arrangements of our Journal. Details on most points are so absolutely necessary, that it is impossible for us to give an abstract of the whole that would be of any important degree of utility; we shall, therefore, only occupy ourselves with that which will be interesting to readers possessed of the elementary information above alluded to; and we think we may thus convey to them the chief of the knowledge appertaining more properly to medical jurisprudence that is contained in this work. We shall confine ourselves to a simple analysis of the parts we take into consideration, as the only conduct by which we shall be able to fulfil our intentions of giving a comprehensive view of what is expressly the result of the author's own researches.

The author commences by remarking, that "the physician, consulted by the magistrate on a case of poisoning, should always have the sentence of Plenck present to his mind: *Unicum signum certum dati veneni est notitia botanica inventi veneni vegetabilis, et analysis chemica inventi veneni mineralis.* This author should, however, have added,—*seu notitia zoologica inventi veneni animalis.* Thus, to affirm that there has been poisoning, it is necessary to demonstrate the presence of the poison, by effecting the chemical experiments proper to render its existence clearly obvious, if it appertain to the mineral kingdom; and by proving its botanic or zoologic character, if it make part of the organic kingdom. But the solution of this problem may be opposed by a multitude of difficulties: on the one hand, the well-known poisonous substances are in very great number; the researches requisite to determine their nature are often very delicate, especially when these substances are combined with matters which mask or decompose them: on the other hand, the poisoning may be the consequence of absorption of the deleterious substance, which is then often inaccessible by all our means of investigation. Sometimes, even, though the poison have not been absorbed, the quantity we can examine is but very small, which increases the difficulty of the process; and, lastly, diseases simulating poisoning by their symptoms, and by the alterations of texture they produce, frequently concur to render more difficult the solution of this question."

Having thus shown the character of the requisite evidence, and the difficulties of attaining it, the author adduces a series of "*preliminary notions on poisoning, considered in a medico-legal point of view.*" He says,—

"The term *poisoning (veneficium, toxicatio,)* is applied to the sum of the effects produced by poisons applied to one or more parts of the bodies of animals. This word is also employed to designate the *act of poisoning*. The word *poison (toxicum, venenum, virus,)* has been by turns defined *a cause of diseases; an agent capable of occasioning a more or less violent death, when introduced into the stomach; all bodies deleterious to the health of man, the action of which is not mechanical*, &c. The following definition, borrowed from Gmelin, appears to me to be preferable. We should consider as a poison a body which destroys health, or entirely annuls life, when it is taken internally or applied in any way to a living body, and in a very small dose.

"Poisons exist in the three kingdoms of nature: it is this which has suggested the idea of arranging them in three classes,—that is to say, *mineral poisons; vegetable poisons;* and *animal poisons.* We think it advisable to adopt the following classification:—1°, *irritative poisons;* 2°, *narcotic poisons;* 3°, *narcotico-acrid poisons;* 4°, *septic poisons.* Certainly this classification, the idea of which is derived from Vicat, is far from being exempt from objections; but, in the present state of science, it appears to me to be preferable to any other."

The *irritative poisons* are, "Phosphorus, iodine, concentrated mineral and vegetable acids, chlorine, eau de javelle (composed of chlorine and potass), potass, soda, lime; sulphuret of potass, nitrate of potass, subcarbonate of potass; barytes, subcarbonate of barytes, hydrochlorate (muriate) of barytes; liquid ammonia, subcarbonate of ammonia, hydrochlorate of ammonia; preparations of mercury, tin, arsenic, copper, silver, antimony; emetine, (the alkaline principle of ipecacuanha;) preparations of bismuth, gold, zinc, iron, and lead; bryony root; elaterium; colocynth; gamboge; daphne gnidium; ricinus communis and inermis; euphorbium; savin; stavesacre; gratiola; anemone pulsatilla; the rhus radicans and toxicodendron; chelidony; the narcissus poeticus and pseudo-narcissus; ranunculus acris; cantharides; certain fish, &c."

The *narcotic poisons* are, "Opium, morphine, and the salt of Derosne (narcotine) hyosciamus; prussic acid; cherry laurel; amygdalus persica; prunus padus; lactusa virosa, ivy, &c."

The *narcotico-acrid poisons* are, "Squill, scillitine, (the alkaline principle of squill;) œnanthe crocata; aconitum napellus; black and white hellebore, veratrine, (the alkaline principle of white hellebore, colchicum autumnale, and sabadilla;) colchicum; belladonna; datura stramonium; digitalis purpurea; conium maculatum; æthusa cynapium; cicuta aquatica; nerium oleander; ergot rye; nux vomica; faba sancti ignatii; upas tieute; the false angustura; ticunas; woorara; curara; camphor; cocculus indicus; upas antiar; poisonous champignons; alcohol, ether, and spirituous liquors in general."

The *septic poisons* are, "Hydrosulphuric acid; venomous animals, as the viper, rattle-snake, scorpion, &c.; putrid matters."

Besides these, the author treats, in a distinct article, of the gases which exert a deleterious action when introduced into the respiratory canals: these gases are the carbonic acid gas, the vapour of burning charcoal, unrenewed air, the gas of privies, ammoniacal gas, azote, chlorine, hydrogen; arseniated and carbonated hydrogen, nitrous acid, protoxyd of azote, sulphurous acid."

Pursuing his general considerations on poisoning, the author says—

"All the poisons do not act with the same energy; there are some which, being administered in a very small dose, produce the death of man and the most robust animals almost instantaneously, (pure prussic acid, upas tieuté, strychnine;) others, on the contrary, do not manifest their agency until after the lapse of a certain time, even when administered in a very large dose,—such are the sulphate of zinc, sedum acre, &c. There are some which may be classed between the two extremes just designated, in regard to their intensity,—such are the nitrate of silver, colocynth, &c.

"The action of poisons varies according to their more or less minute division: in general, all other circumstances being equal, they act more powerfully in proportion as they are more divided; consequently, the effects produced by a substance dissolved in water should be more intense than those caused by the same substance in a pulverulent state.

"When poisons are introduced in the intestinal canal, their action will be greater, other circumstances being similar, in proportion as the canal approaches more to the state of vacuity.

"Substances capable of poisoning man do not act in a similar manner on all species of animals: we may nevertheless establish, without fear of being in error, that what is poisonous to man is equally so to dogs; though it is often necessary to administer a stronger or a weaker dose, to produce a given effect in the latter, than that which is sufficient to cause the same effect in the former. Authors who, in opposition to this proposition, have stated that oxyde of arsenic, the very deleterious effect of which on man is so generally known, acts on dogs only as an hypercathartic, have evidently been deceived: and it results from this fact, that the study of poisoning in man may be favoured, in a very important degree, by experiments made on dogs. The jurisprudential view of this subject has been particularly indebted, for the progress it has lately made, to chemical experiments on the matters contained in the digestive canal of dogs killed by poisoning.

"It is not necessary that poisons should be introduced into the stomach by the mouth, in order that their injurious effects may be developed. Several of them occasion their proper consequences when passed into the rectum, as a glyster. Some of them act with energy when they have been applied to the mucous membrane of the mouth, nostrils, eye, and vagina. There are some of them which, on being merely placed in contact with the skin, will produce there inflammation and suppuration, and at length all the symptoms of poisoning. The same phenomena are evinced when they are applied on the subcutaneous laminated tissue. This effect may also be the effect of the application of a plaster, or any other form of external application by frictions, in the composition of which there enters some poisonous substance. But it is especially when certain poisons are applied to the serous and venous textures, that we remark the full energy of their action.

"The agency of poisons on man varies according to the nature of those substances. There are some which irritate, inflame, and destroy the parts on which they have been applied, and then produce effects which may be regarded as sympathetic. Others act but imperceptibly, or apparently not at all, on the texture with which they are in contact, but appear to be absorbed; they are carried into the torrent of the circulation, and then proceed to exert their deleterious influence on the nervous system, the organs of the circulation, respiration, digestion, &c.

"The absorption of certain poisons appears to be demonstrated. Whilst we wait in order that new researches may sufficiently instruct us respecting this function, we think it may be established: 1°, that substances susceptible of absorption are absorbed, in general, with more energy when dissolved in water than when in a pulverulent state; 2°, that the insolubility of substances does not always prevent their absorption; 3°, that this function is not exerted with the same energy in all the different tissues; that it is more powerful in the serous than in the mucous tissue and, for stronger reasons, the subcutaneous laminated texture; 4°, that, though a substance applied to the exterior of the body is generally absorbed more promptly in proportion as the part is more abundantly supplied with venous and lymphatic vessels, the contrary is, nevertheless, sometimes observed; 5°, that there is reason for believing that when a poison of the vegetable kingdom, composed of several immediate principles, is absorbed, it is not entirely absorbed; but that a decomposition of it is effected, one certain principle being absorbed whilst another is not.*

"It is not always easy to judge whether or not a poisonous substance has been absorbed. It is important, however, in certain cases of legal investigation, that this point should be determined. The following precepts may be considered of general application.

"If the application of a poison to the subcutaneous cellular texture has not given rise to any sign of local irritation, when the patient has died shortly after it, and when, on dissecting the body, we find alteration of structure in the lungs, heart, and digestive tube, it may be considered that the poison has been absorbed. This inference acquires much more validity if, on placing the poison in contact with diverse tissues, we find that it constantly produces similar phenomena, and that death arises proportionally more prompt as the part to which it is applied is endowed with a greater degree of absorbent power. We may, on the contrary, affirm that absorption has not taken place, when we witness, after the exterior application of a poisonous and irritating substance, only phenomena similar to those which arise from a burn of moderate extent.

"There are solid, liquid, and gaseous poisons. The last are often the source of very serious difficulties to the practitioner who is required to give evidence on a case of poisoning: indeed, it is possible that the

* "Some physiologists have expressed doubts of the absorption of vegetable substances. We should, they say, be able to demonstrate the presence of the poison in the organ on which it has acted, that we may be certain it has been absorbed. They explain the phenomena which we refer to absorption by the action of the nerves."

subject has been made to inspire an irritating or septic gas, the presence of which cannot possibly be ascertained after death. Sometimes, however, the nature of the gas may be strictly known; for example, when the patient has been suffocated in an insalubrious atmosphere, and it is possible to submit this atmosphere to chemical experiments. In general, it is much easier to discover the poison when it is solid or liquid; the difficulty is still less when it is a mineral substance. The following precepts relative to poisons of an inorganic kind should always be kept in view: 1°. The solid or liquid poisons, in question, administered without having been mingled with any other body, may not have been wholly used; the practitioner may then easily recognize them, by submitting the remaining portion to proper chemical examinations. 2°. When they have been mingled with other poisons, or with solid or liquid alimentary matters, and they have not been entirely used, we should, to discover them, most frequently have recourse to chemical experiments of another kind, which will be hereafter described. 3°. When we cannot procure any remains of the poison, we must, necessarily, analyze the matters vomited or evacuated by stool; and, when the patient has died, it will be necessary, when the poison has not been discovered in the matters contained in the cavity of the intestines, to submit the textures of this canal to particular experiments, the principal object of which is the destruction of their organization, by which the poison, when it exists, may be obtained in an isolated manner. 4°. The chemical means employed in the solution of the question under consideration are qualified to render manifest the smallest quantities of the mineral poisons."

From these preliminary remarks, the author proceeds to treat of "poisons in particular," and first of the irritative poisons. "The term *irritative*, *corrosive*, *escharotic*, or *acrid*, poisons," he says, "should be applied only to those the effects of which are the result of the irritation and inflammation they produce in the parts of the body to which they are applied, and which may, ultimately, give rise to ulceration and eschars: in this case, several poisons ranged in the class in question should be placed elsewhere, since they destroy life in a very short space of time, leaving hardly any traces of their local action."

The *symptoms and lesions of tissue produced by* PHOSPHORUS have the closest analogy with those which are the result of the introduction of the mineral acids into the stomach: indeed, phosphorus is transformed, in the digestive canal, into either phosphoric or phosphatic acid, according as it absorbs more or less oxygen from the air contained in this canal. If it has been administered in a pulverulent form, or, still more, dissolved in ether, in oil, &c. it passes to the state of phosphoric acid, and it acts as an energetic irritant: if it has been introduced in a solid state, and under the form of cylinders, it is converted into phosphatic acid, which is much less active.

The *symptoms* produced by IODINE are, also, similar to those caused by the mineral acids; but, in the *lesions of tissue* resulting from its agency on the intestinal tube, there is something peculiar. The mucous membrane of the stomach presents small linear ulcers bordered by a yellow aureola: the ulcerated portions are pellucid; we see here and there, on the interior surface of the organ, and principally on the folds which are adjacent to the pylorus, several spots of a bright-yellow colour; sometimes the colour of these spots verges to brown; the mucous membrane may be very easily detached in these places; we often find the mucous membrane near the pylorus red, inflamed, and covered with a deep-green coating, which prevents our discovering, at first, the redness of the parts beneath it.

The action of iodine on the animal economy "appears to be the same," according to the author, "with that of other irritants which are not absorbed." This mineral acts only after it has been transformed into hydriodic acid at the expence of the aqueous fluids or the tissues of the animal subjected to its application.

The symptoms of poisoning by the powerful acids,—as the sulphuric, nitric, nitrous, hydrochloric (muriatic), aqua regia, phosphoric, phosphatic, oxalic, tartaric, and citric,—are as follows:

"Immediately after the acid has been swallowed, a burning, very disagreeable, acid taste; a sense of acrid heat in the throat and stomach; acute pain about the fauces, that very soon extends to the bowels; intolerable fetor of the breath; frequent belchings; inclination to vomit, and expulsion by vomiting of matters variable in colour, sometimes mingled with blood, producing a sense of bitterness in the mouth, very often causing effervescence with chalk, and reddening the tincture of turnsole, like all the acids; hiccup; sometimes constipation of the bowels, but most frequently copious, and more or less bloody, stools; acute pains throughout the whole of the belly, which extend even into the chest; difficulty of breathing; sense of great anguish; frequent and irregular pulse; ardent thirst, whilst liquids drank increase the pain and are soon vomited; shiverings from time to time, and almost always the skin, and especially that of the lower limbs, is icy cold; cold and clammy sweats; repeated and fruitless efforts to void the urine; impossibility of keeping in one position; convulsive motions of the lips, face, and limbs; great prostration of strength: the physiognomy is but little altered at first, but the countenance soon becomes pale and of a leaden hue. The integrity of the intellectual faculties is most frequently preserved. It is not rare to see the interior of the mouth and lips burned, thickened, and almost covered with white or black spots, the detachment of which produces irritation and a fatiguing cough; the voice then becomes altered. There is sometimes a painful eruption on the skin. The whole of these symptoms are not constantly observed in the same individual."

The *lesions of tissue* produced by the concentrated acids, that are common to the whole of them, are thus described by the author:

"When they are received into the digestive canal, they inflame all the parts they touch; the inflammation is, in general, but slight where the acid has rapidly glided over the part, and more or less intense where it has rested for some time; thus, the different parts of the mouth, the pharynx, and the œsophagus, are ordinarily the seat of a more or less intense degree of redness; the stomach and intestines present most frequently signs of more violent disorder. Sometimes the mucous membrane lining these organs is of a vivid red, or cherry-red, or a brownish-red, colour, throughout its whole extent: in this case, the subjacent muscular and serous tunics may participate, in a less degree, in the inflammation. Sometimes we observe, besides this general redness, blackish spots dispersed here and there on the interior surface of the stomach; these spots, real ecchymoses, are formed by blood extravasated in the aureolas of the cellular tissue situate beneath the mucous membrane, and should be distinguished from gangrenous patches. Sometimes we find proper eschars, and ulcers which may interest the whole of the tunics. In certain cases, the tissues are thickened; in others, they are softened, and as it were dissolved, so that the membranes may be detached with the greatest facility. There are cases where the stomach and rectum are found much inflamed, whilst the small intestines are almost in the natural state: this circumstance, which happens from a great number of poisonous substances, appears to depend on the rapidity with which the poison traverses the small intestines, and the long stay it makes in the stomach and rectum."

Their action on the animal economy is thus described: "1°. The concentrated acids act with the greatest energy when they are introduced into the digestive canal; the death which they produce is the result of the inflammation they develop in the tissues of this canal, and of the sympathetic irritation of the brain and the nervous system. 2°. They are not absorbed. 3°. Injected into the veins, they coagulate the blood, and instantaneously destroy life. 4°. Applied to the skin, they give rise to the phenomena of a burn, and they occasion death only when the parts are thus affected to a certain and considerable extent."

On treating of the *sulphuric acid* in particular, the author remarks that, besides the alterations of tissue already described as common effects of the acids in general, we observe that this acid, when in a concentrated state, "often reduces many of the parts it touches to a sort of black pulp." *Composition* blue (consisting of indigo and the acid,) produces the same effect; only we find here and there, in some parts of the digestive canal, a greenish or yellowish tint.

The acid may be introduced into the alimentary canal after the death of the subject, for certain purposes, and the knowledge of this fact may become of serious import in the event of a forensic investigation. The author has not neglected the means of acquiring precise information relative to this circumstance: the results of his experiments have shown, that "when five or six drachms of concentrated sulphuric acid have been introduced into the rectum of an individual soon after death, and it has been permitted to remain there for four-and-twenty hours, we remark, on opening the body, that the acid has acted only on the *portion of intestine to which it has been applied*, so that there is a well-defined line of demarcation between the parts which have, and those which have not, been touched by the acid. The mucous membrane is yellowish, and may be easily detached in a flocculent form; the muscular tunic is white; the peritoneal coat is also white, but is, besides, thickened and interwoven with hardened vessels, looking as if they had been filled with a black injection, or as though the blood they contained had been carbonized by the acid: *we discover no trace of redness.* These characters are quite sufficient to enable us to distinguish whether the acid has been applied before or after death.

The *nitric acid,* besides the symptoms common to the effects of the acids in general, produces often yellowish, citrine, or orange-coloured spots, on the chin, lips, and the hands. The sense of cold which it causes in the patient is very marked, and persists for a long time.

The *lesions of tissue* especially resulting from its action are, 1°, a whitish, or more frequently yellowish, tint of the mucous membrane of the mouth and œsophagus, and of the crown of the teeth; 2°, a very thick crust, of a greenish-yellow colour, on the internal surface of the stomach, duodenum, and jejunum: this latter appearance is, however, far from being constant, for, very often, the vivid redness which characterizes inflammation of the membranes of the stomach and two first portions of the small intestines, has succeeded to the yellow hue which the nitric acid had occasioned in the first moments of its action; besides this, other acids than the nitric, as the sulphuric and muriatic, may, under certain circumstances, give a yellow tinge to the internal membrane of the duodenum: a phenomenon dependent on the decomposition of the bile contained in this intestine, and of the application of part of the yellow matter of this fluid to the interior surface of the organ. When half an ounce of the nitric acid of commerce has been injected into the rectum of a person after death, and the body is opened after the lapse of four-and-twenty hours, we observe all the tunics of the portion of intestine with which the acid has been in contact tinged of a bright-yellow colour; the mucous membrane is sometimes destroyed, and transformed into a flocculent substance of a light-yellow colour and greasy aspect: *no trace of redness or of inflammation is present.* If the acid has remained for a long period of time in the alimentary canal, the lesion is carried still further; for the intestine may be reduced, on being rubbed between the fingers, into a sort of fatty paste, of a very clear yellow colour."

The symptoms produced by potass, soda, and lime, hardly differ, the author says, from those resulting from the concentrated acids: "it is only necessary to notice, that the taste of these poisons is acrid, caustic, and urinous, and that the matters vomited, instead of being acid and causing effervescence with chalk, are alkaline, and render green the syrup of violets." He refers to what has been said respecting the acids, for the *lesions of tissue* produced by the alkalies, without describing any thing peculiar to the latter.

The *sulphuret of potass,* (or of potassium,) and the *subcarbonate* and *nitrate of potass,* are said to produce symptoms which "have the greatest analogy with those developed by the acids: however, it should be observed,—1°, that the matters vomited do not cause effervescence with chalk; 2°, that the taste experienced by the patient, instead of being acid, is acrid, caustic, bitter or saline, and somewhat pungent." For the *lesions of tissue*, in general, we are referred to those attributed to the acids. There are these circumstances, however, particularly noticed in regard to the sulphuret of potass, that, instead of acting as the common alkaline irritants, it, under certain conditions, acts particularly by the hydrosulphuric acid which is disengaged from it in the stomach, and then it gives rise to the symptoms described or referred to in the following paragraph.

"1°. The liver of sulphur, when introduced into the stomach of men and dogs, acts in the manner of irritative poisons, (as described in the effects of the concentrated acids,) and may produce death in the space of a few hours, when it has been administered in the dose of a few drachms, in the solid state or in a strong solution, and has not been rejected by vomiting soon after its reception into the stomach; 2°, it is decomposed when the stomach into which it is introduced contains a *large quantity of free acid,* as is sometimes the case; and then death may be the result of hydro-sulphuric acid gas (sulphureted hydrogen,) which has been evolved: in this case, the interior of the stomach is lined with a layer of sulphur, and we find, in the different organs and in the blood, the alterations we shall notice when we treat of the hydrosulphuric acid; 3°, when, on the contrary, the quantity of free acid contained in the stomach is inconsiderable, *which is most frequently the case,* the deleterious effects of this mineral cannot be attributed to the sulphureted gas which is disengaged, the quantity of this gas being less than that which man suffers daily with impunity; so death does not happen until after the lapse of four-and-twenty or six-and-thirty hours, (when one or two drachms of the sulphuret has been administered,) and the alterations of the organs and fluids, far from being the same with those produced by the hydro-sulphuric acid, resemble entirely those which arise from irritants; 4°, we should deceive ourselves if we were to infer that, because death has taken place within a few minutes after the ingestion of a large dose of the sulphuret, such an event is the result of asphyxia produced by the sulphureted hydrogen gas; for several of the poisons of the class of irritants, in which we find neither sulphureted hydrogen nor the elements proper for forming it, act, in this respect, in the same way as the liver of sulphur, when they are administered in large doses."

The following are the author's observations respecting the lesions of tissue produced expressly by the sulphuret of potass.

"When there has been introduced into the stomach a dose of liver of sulphur sufficiently strong to produce death, we observe different lesions of the digestive tube, according to the duration and intensity of the poisoning:—1°, sometimes the internal membrane,—of a vivid red colour throughout its whole extent, or in several points,—is lined by a layer of sulphur, of a greenish-yellow colour, thick, and readily removeable; the redness and the coating just described are sometimes found in the intestines. 2°. Sometimes the interior of the stomach is rugous, of a deep-green colour, interspersed by yellowish-white spots; the internal membrane of the viscus which is the seat of these alterations, is covered with sulphur; the muscular tunic is of a brownish-red hue in its interior part, and green at the surface which is immediately in contact with the serous membrane; ecchymoses, of more or less considerable extent, are observed between the mucous and muscular coats, and correspond exactly with the yellowish-white spots which have just been noticed; the small intestines are the seat of more or less intense inflammation. 3°. Sometimes *it is impossible to discover the smallest layer of sulphur* in the interior of the digestive tube: the mucous membrane of the stomach, of a vivid red colour, presents several large and circular ulcers, between which are spots of ecchymosis of various dimensions. The lungs, ordinarily slightly crepitating, are in some cases soft and gorged with livid, black, and extremely fluid blood; in other cases, they are hard and contain but little air. The left ventricle of the heart, if examined immediately after death, contains, in certain circumstances, more or less of black blood."

With respect to the action of the *nitrate of potass* on the animal economy, the author says, "it results from a great number of experiments on dogs, and of several observations made on man, 1°, that this salt, when introduced into the stomach, is poisonous, and capable of causing death in the space of a few hours, *when it is not vomited, and has been administered in the dose of a few drachms, in powder or in a strong solution*; 2°, that it causes inflammation, ordinarily very intense, of the tissues of the digestive canal, followed by affections of the nervous system, having the greatest resemblance with those produced by stupefying poisons; 3°, that it is not absorbed when applied to the subcutaneous cellular tissue; and, consequently, that its immediate effects in this case are merely local; 4°, that its action differs from that of the neutral salts employed as purgatives."

---

